# Monkeypox virus emerges from the shadow of its more infamous cousin: family biology matters

**DOI:** 10.1080/22221751.2022.2095309

**Published:** 2022-07-12

**Authors:** Yan Xiang, Addison White

**Affiliations:** Department of Microbiology, Immunology and Molecular Genetics, University of Texas Health Science Center, San Antonio, TX, USA

**Keywords:** Poxvirus, monkeypox, host range, evolution, antibodies

## Abstract

Monkeypox virus (MPXV) is closely related to the infamous variola (smallpox) virus, causing a febrile rash illness in humans similar to but milder than smallpox. In the twentieth century, human monkeypox had been mostly a rare zoonotic disease confined to forested areas in West and Central Africa. However, the case number and geographic range have increased significantly in this century, coincided with the waning of the smallpox vaccine-induced immunity in the global population. The outbreak of human monkeypox in multiple countries since May 2022 has been unusual in its large case number and the absence of direct links to endemic countries, raising concerns for a possible change in monkeypox transmission pattern that could pose a greater global threat. Here, we review aspects of MPXV biology that are relevant for risk assessment and preparedness for a monkeypox epidemic, with an emphasis on recent progress in understanding of the virus host range, evolutionary potential, and neutralization targets.

## Monkeypox virus classification

Monkeypox virus (MPXV) is a member of the Orthopoxvirus (OPXV) genus of the *Poxviridae* family [1]. Poxviruses are large, enveloped viruses. Their genome consists of a linear, double-stranded DNA (dsDNA) of ∼200 kilobase pairs, closely packed with ∼200 genes. Approximately half of the genes are well conserved amongst vertebrate poxviruses and are essential for viral replication, while the remaining half are so-called “accessory” genes that are mostly involved in virus-host interactions and may individually be dispensable for viral replication [2].

The OPXV genus has more than 10 member species [3], including variola (smallpox) virus (VARV), vaccinia (the smallpox vaccine) virus (VACV), cowpox virus (CPXV), camelpox virus (CMLV), and several novel species isolated from infected humans or primates since 2010 [4–6]. OPXV species are often named after the host from which they were initially isolated, but they are all believed to descend from a rodent-borne ancestor, and some of them, including MPXV and CPXV, still use rodents as the reservoir hosts. OPXV species are genetically and antigenically closely related; the immunity against one species cross-protects against other species. Their major differences lie in host range and virulence. MPXV, CPXV and VACV can infect a broad range of mammalian species, while VARV and CMLV only have one known host. CPXV causes a mild disease, while VARV famously caused smallpox with up to 30% case fatality.

MPXV strains are divided into two clades that show ∼0.5% genomic sequence difference and circulate in different regions of Africa [7,8]. The Congo Basin (central African) clade is more virulent than the West African clade in humans and cynomolgus monkeys [7]: their respective human case fatality rates are estimated to be 10.6% or 3.6% [9].

## History and trend of monkeypox outbreaks

Readers are referred to a recent literature review for the detailed statistics of monkeypox cases prior to 2019 [9]. Case statistics after 2019 are available in World Health Organization publications including the weekly bulletin from WHO Africa Regional Office (AFRO) [10].

### Discovery

MPXV was first isolated in Copenhagen, Denmark in 1958 during two outbreaks of a nonfatal rash disease among captive cynomolgus macaques imported from Singapore [11]. In the subsequent decade, several similar outbreaks in primate colonies in Europe and the U.S. were reported [12]. It was not until 1970 that the first human case was discovered and MPXV recognized as a human pathogen. The first case of a child in the Democratic Republic of the Congo (DRC) was identified through the intense surveillance for smallpox-like diseases in West and Central Africa during the late phase of the worldwide smallpox eradication campaign [13,14]. Shortly afterwards, six additional cases were identified in Liberia, Nigeria, and Sierra Leone [15].

### A worsening epidemic in Central Africa and re-emergence in West Africa

For the next thirty years after the initial discovery, < 1000 laboratory-confirmed human monkeypox cases were reported worldwide; the vast majority of them (∼96%) were in DRC (Central Africa) with the rest scattered in seven other Central or West African countries. In the twenty-first century, however, the case number has increased dramatically, and their geographic range has expanded. The incidence rate in DRC was shown to have increased 20-fold between the 1980s and mid-2000s by one study [16]. During the decades of 2000–2009 and 2010–2019, suspected cases reported in DRC were >10,000 and >8,000, respectively. From 2020 to May 2022, 10,545 suspected cases and 362 associated deaths have been reported in DRC [10]. The Republic of Congo had its first human monkeypox outbreak in 2003 [17], while South Sudan (East Africa) had its first in 2005 [18]. After 3 or 4 decades of absence, human monkeypox cases re-emerged in the Central African Republic, Sierra Leone, Liberia, Cameroon, and Nigeria (detailed below) in 2010–2019.

In Nigeria (West Africa), only three confirmed monkeypox cases were reported in the 1970s, with the last one in 1978. In September 2017, however, human monkeypox re-emerged after a hiatus of 39 years. Cases have been reported annually ever since, making the outbreak the largest of the West African clade of MPXV to date [19]. As of April 2022, there has been 558 reported cases. Eight associated deaths were reported, some of which were among HIV-infected individuals [19]. While previous outbreaks occurred almost exclusively in rural villages around the forested areas of Africa, the current Nigeria outbreak involves many people living in urban areas [19].

### An Emerging threat outside of Africa

In 2003, the geographic range of human monkeypox expanded outside of Africa for the first time, when 47 cases were identified in the Midwest states of the U.S. [20]. The U.S. outbreak was traced back to Gambian pouched rats (Cricetomys gambianus) imported as exotic pets from Ghana (West Africa). The imported rodents transmitted MPXV to prairie dogs when they were housed together at a pet distribution centre, and the prairie dogs in turn transmitted the virus to their owners. The virus isolated in the U.S. cases belongs to the West African clade. Notably, monkeypox has never been reported in Ghana where the infected rodents originated, suggesting that monkeypox cases are under-detected in Africa.

From September 2018 to November 2021, sporadic human monkeypox cases were reported in several non-African countries, and they were all associated with travels to Nigeria: seven in the United Kingdom, one each in Israel and Singapore, and two in the U.S. [21]. Except for one case in U.K. [22], human-to-human transmission was not detected.

Since 7 May 2022 and as of this writing (9 June 2022), 1,273 confirmed cases have been reported in 31 countries outside the monkeypox endemic regions [23]. Most of the cases do not have a direct link to travel to endemic countries and mainly involve individuals identified as men who have sex with men [24,25]. The viruses isolated from the cases so far all belong to the West African clade [24,25].

## Clinical features

### Transmission

Monkeypox in Africa is typically a zoonosis involving contacts with animals or their bodily fluids, respiratory droplets, and lesion materials. The point of contact is almost always speculated, but dead animals and fomites are also of suspicion as OPXV virion is remarkably stable, an extreme example being the isolation of infectious virus from smallpox scabs after 13 years of room temperature storage [26]. Human-to-human transmission occurs also through close contacts, with an estimated secondary attack rate of ∼9.3% for smallpox-unvaccinated individuals [27]. The current monkeypox outbreak amongst many men who have sex with men raises concerns about possible sexual transmission [28].

### Symptoms

Human monkeypox is similar to but milder than the now-extinct smallpox, with three distinct phases: incubation, prodrome, and rash [29]. The incubation phase ranges from 7 to 14 days, with an average of 13 days [19,29]. The prodrome phase typically includes fever and lymphadenopathy [29], the latter being a feature that distinguishes monkeypox from smallpox and chickenpox. The rash follows a distinct pattern of development: starting with a macular rash and progressing through papular, vesicular, and pustular stages before crusting over and falling off. Rash is mostly on the face, trunk, and extremities, but may involve other areas including the genitalia [19]. Rash lesions on the body are all in the same stage of development, a distinguishing feature of monkeypox and smallpox from other more common rash illnesses such as chickenpox [29]. The lesions contain infectious virus that can be transmitted through direct contact. Secondary complications of infection include bacterial skin infection, gastroenteritis, sepsis, bronchopneumonia, encephalitis, and keratitis [30]. Clinical sequalae associated with post-monkeypox resolution can include hyper- and hypo-pigmented atrophic scars, patchy alopecia, hypertrophic skin scarring, and contracture/deformity of facial muscles following healing of ulcerated facial lesions [19,30,31]. The Congo Basin clade is associated with more severe illness and higher mortality rate than the West African clade. Patients with HIV are more likely to develop secondary bacterial skin infections and are associated with higher mortality [19].

## Virus host range

How MPXV is maintained in nature and, more broadly, what determines the host range of poxviruses are not well understood, although significant progress has been made in the past decade on addressing the latter question. Additional insights on these questions are necessary for the control of MPXV zoonosis in endemic areas and for preventing the introduction of MPXV to new reservoir hosts outside the endemic areas.

### MPXV host range

Current data from virus isolation and serological survey suggest that certain sylvatic rodent species, particularly African rope squirrels, might be the MPXV reservoir hosts, while primates are the incidental hosts. MPXV has been isolated only twice from wild animals despite considerable efforts: once from a rope squirrel in DRC in 1985 [32] and once from a sooty mangabey in Taï National Park of Cote d’Ivoire in 2012 [33]; the animals were ill or dead with pox-like lesions. Further insights largely came from field surveys of anti-OPXV seroprevalence among wild animals, with the notable caveat that OPXV seropositivity could also be due to past infection by other, perhaps unidentified OPXV species. In some areas of Ghana and DRC, rope squirrels (Funisciurus), dormice (Graphiurus), African giant pouched rats (Cricetomys), and sun squirrels (Heliosciurus) were found to have the highest frequency of OPXV seropositivity, with some animals also having evidence of OPXV DNA in tissues [34–36]. Lower frequencies of anti-OPXV were found in a few other rodent species (rufous-nosed rat, striped mouse and gerbil) and elephant shrew [34–36]. Additionally, anti-OPXV antibodies were found in several non-human primate (NHP) species in West and Central Africa, while they were absent in large cohorts of wild NHPs in other parts of the world [37]. In recent years, repeated occurrences of monkeypox outbreak were reported among chimpanzees housed at wildlife sanctuaries in Cameroon and Cote d’Ivoire [38,39].

Under laboratory or captive conditions, a very broad range of mammalian taxa were found to be susceptible to MPXV infection [40], including rabbits, ant-eaters, opossums, and additional rodent species such as prairie dogs and ground squirrels. It is worth pointing out that ground squirrels that are abundant in grassland of North America are highly susceptible to MPXV [41], raising concerns about MPXV establishing reservoirs in North America rodents due to human spillbacks to animals. It is postulated that the maintenance of MPXV in nature may depend on its ability to utilize multiple host species [40].

### Poxvirus host range genes

Poxvirus host range is not impacted by the cell entry step like many other viruses are. Rather, poxviruses can enter nearly all mammalian cells, and their host ranges are mainly determined by their abilities to circumvent host antiviral responses, which may display host species-specific differences. Divergence in host range of different poxvirus species is at least partially due to difference in the repertoire of around ten viral accessory genes, which were empirically defined as host range genes and subsequently found to inhibit different aspects of cellular innate immunity [42–44]. Significant progress has been made in the past decade on research of poxvirus host range genes, including discovery of additional viral host range genes, identification of their host targets, and uncovering the co-evolution of the viral genes and their host targets, which altogether shed light on how poxvirus host range is determined. Some key research findings are described below.

The cellular antiviral factor, Protein kinase R (PKR), has been recognized as a critical barrier for poxvirus replication, and it is targeted by two poxvirus host range genes, E3L and K3L (VACV gene name). Primate PKR genes were found to have undergone rapid evolution and show difference in susceptibility to K3L inhibition[45,46]. More broadly, divergences of PKR genes of a wide variety of mammalian species were found to result in different susceptibility to the K3L orthologs from various poxviruses, which in turn inhibit PKR in a species-specific manner [47,48].

Mammalian Sterile Alpha Motif Domain-containing 9 (SAMD9) [49,50] and its paralog, SAMD9L [51], were recently found to be host restriction factors for poxviruses. Together, SAMD9 and SAMD9L (SAMD9/9L) form a crucial host barrier that poxviruses must overcome for infection and pathogenesis [51]. They are targeted independently by three distinct poxvirus host range genes, K1L, C7L, and CP77. SAMD9 and SAMD9L (SAMD9/9L) genes show significant divergence across mammalian species particularly among the rodents [52]. Species-specific difference in SAMD9/9L were found to have resulted in different susceptibility to poxvirus host range gene products [51,53]. Particularly, SAMD9L from an old-world rodent (Chinese hamster) is resistant to both K1L and C7L, and only susceptible to CP77, rendering the Chinese hamster cells nonpermissive to poxvirus species that do not encode CP77 [53]. Notably, K1L and/or CP77 are lost from OPXV species with a narrow host range (VARV and CMLV) while maintained in MPXV, a feature that was speculated to be important for broad host range OPXVs to overcome diverse SAMD9/9L in rodents [53].

The C12L and C16L genes were recently identified as the missing host range genes in Modified vaccinia virus Ankara (MVA) that render MVA replication incompetent in human cells [54], making MVA a safer smallpox (and monkeypox) vaccine. The host range function of C12L is not completely understood [55]. C16L functions by inhibiting human Zinc-finger antiviral protein (ZAP), which was found to be a host restriction factor for MVA [56].

## Poxvirus evolution

A major concern for zoonotic pathogens is evolution towards more transmissible or virulent in humans. A specific fear regarding OPXVs such as MPXV is the possibility of them becoming pathogens capable of starting another smallpox-like pandemic. An insight into MPXV evolutionary potential can be gleaned from a review of poxvirus evolution mechanisms, some of which are quite unique amongst viruses.

### Evolutionary rate

Poxvirus has a lower mutation rate than RNA viruses, as its DNA genome is replicated by a viral DNA polymerase that possesses 3’−5’ exonuclease proofreading activity [57]. The substitution rate of poxviruses estimated from molecular clock analysis are in the range of 2 × 10^−6^–1 × 10^−5^ nucleotide substitutions/site/year [58,59], which could result in as much as 2 nucleotide changes in the genome per year. For comparison, the substitution rates for RNA viruses are in the range of 10^–2^–10^–5^ nucleotide substitutions/site/year [60]. It is worth noting that the poxvirus substitution rate is less than two orders of magnitude slower than that for SARS-CoV-2, which was estimated to be ∼6.58 × 10^−3^ subs/site/year by one study [61]. Furthermore, as described below, the large, flexible genome of poxvirus allows large structural changes that result in gene loss or gene gain and more quickly alter viral phenotypes.

### Gene loss during orthopoxvirus evolution and smallpox emergence

Comparative genomics indicate that all extant OPXV species descended from a common ancestor through a process that is dominated by lineage-specific loss of a subset of the accessory genes [2,62]. The ancestral virus is believed to be closest to certain CPXV strains, which possess nearly the full set of the accessory genes and have a broad host range. VARV has the smallest genome among OPXV, and a number of accessory genes were fragmented or deleted. The loss of the accessory genes confined VARV to the human host while counterintuitively making it more virulent in humans. The significance of the reductive evolutionary process in smallpox emergence is supported by the recent discovery of ancient VARV strains in some Viking age (6th to 7th century CE) human remains [63]. The ancient strains were found in a surprisingly large percentage of the human remains and retained some accessory genes that are absent in the modern strains, lending to the speculation that the Viking age strains with the additional accessory genes caused a more widespread but milder human disease than modern-day smallpox [64].

Compared to VARV, MPXV contains a larger number of the accessory genes, and its host range is significantly broader. The current knowledge of poxvirus biology is not adequate for predicting whether the loss of additional accessory genes, and if so, what gene losses, would change MPXV transmission or virulence in humans. However, gene loss events during MPXV evolution should be heeded. Notably, genomic surveillance of endemic monkeypox cases from 2005 to 2007 in West Africa revealed the loss of a specific accessory gene in ∼17% of the samples, seemingly correlating with an increase in human-to-human transmission [65].

### Recombination in poxvirus evolution

Poxviruses can accommodate the insertion of large fragments of foreign DNA in their genomes through recombination, a property that has made VACV a useful vaccine vector. Poxviruses recombine at a high level under laboratory conditions, and some naturally derived poxvirus recombinants have been isolated from nature. A novel CPXV strain isolated from a Norwegian patient has a mosaic genome that may have arisen out of recombination with three other OPXV species [66]. Several poxvirus species isolated in this century from human patients, including two novel OPXV species, show evidence of recombination with OPXV [5,6]. A striking example that illustrates the potential impact of poxvirus recombination on viral transmission is a novel myxoma virus strain isolated in 2019. Myxoma virus is a poxvirus that naturally infected rabbits, but a strain was isolated from ill Iberian hares, and its genome has a fragment from an unidentified, presumably ungulate-associated poxvirus [67]. Importantly, through this recombination, the virus acquired a C7L-like poxvirus host range gene [68], allowing it to jump into a new host species and cause a large, fatal outbreak in wild hares.

### Gene amplification in poxvirus evolution

Gene duplication followed by diversification is believed to have expanded several poxvirus accessory gene families during ancient poxvirus evolution [2]. Gene amplification also appears to be a quite common mechanism for poxvirus to rapidly adapt to environmental stress, at least under laboratory conditions. For example, when passaged in the presence of hydroxyurea or rifampin, drugs that inhibit different steps of VACV replication, drug-resistant VACV mutants quickly emerged [69,70]. Many of the mutants contain in their genome tandem repeats of a viral gene that is either the direct drug-target or a close partner of the drug-target [69,70], allowing them to overcome the drug effect by increasing the gene dosage. Similarly, to overcome the action of human antiviral protein PKR, VACV quickly expanded their genome by duplicating a viral gene (K3L or a similar gene) that encodes a weak human PKR inhibitor [71,72]. Following the gene amplification, a beneficial point mutation emerged in some K3L gene copy, which in turn allowed the subsequent contraction of K3L tandem repeats into one with the point mutation [71]. This multistep evolution process was dubbed “gene accordions” and may speed up poxvirus evolution [71].

## Vaccines

After the 9/11 attacks, to prepare the public in the event of a bioterrorism act with smallpox, the U.S. government led a concerted effort to develop antivirals and next-generation vaccines against smallpox. The goal of developing two antivirals with different mechanisms of action and two vaccines took nearly 20 years to fruition. Consequently, there are now two FDA-approved vaccines and two FDA-approved antivirals against smallpox, which are expected to be effective against monkeypox.

The first next-generation smallpox vaccine is ACAM2000, which is similar to the discontinued Dryvax vaccine, as it is produced on cell culture with a clone of Dryvax. Smallpox vaccines like the Dryvax are known to generate long lasting immunity, with specific antibodies and memory B cells detected more than 60 years after the vaccination [73,74]. Historical data indicate that the vaccine provides 85% protection against human monkeypox [75]. ACAM2000 contains replication competent VACV and is administered by skin scarification. Successful administration produces a take at the vaccination site containing virus capable of transmission through autoinoculation and inadvertent inoculation of close contacts. The vaccine is contraindicated in individuals with pregnancy, atopic dermatitis, or immune deficiencies, among others [76]. It is expected to have a similar safety profile as the Dryvax vaccine, which is known to be associated with some serious adverse events. Myopericarditis has been reported among some vaccinees [28].

The second next-generation smallpox vaccine is MVA-BN (JYNNEOS in the U.S.), which is manufactured with the Modified vaccinia Ankara strain (MVA). MVA is replication-impaired in most mammalian cells, partly due to the loss of two host range genes as described in the previous sections. The MVA-BN vaccine is administered by two subcutaneous injections 4 weeks apart and does not produce a take [76]. No serious adverse events are expected, and there is no risk for autoinoculation and inadvertent inoculation. The vaccine is approved in the US for use against both smallpox and monkeypox based on its immunogenicity in clinical studies as well as efficacy data from animal challenge studies, but human efficacy has not been proven with clinical trials.

## Antiviral drugs

ST-246 (Tecovirimat) and Brincidofovir are two antivirals that have been approved in the U.S. for treating smallpox. ST-246 targets a highly conserved OPXV envelope protein (F13L) and inhibits virion release. Brincidofovir is an orally bioavailable lipid conjugate of cidofovir, an acyclic nucleoside analog that is licensed for treating human cytomegalovirus infection. The mechanism of action of cidofovir is inhibition of poxvirus DNA replication. The use of the drugs in a limited number of human monkeypox cases suggest tecovirimat is effective while brincidofovir has poor efficacy [77]. When passaged in the presence of either ST-246 or cidofovir in cell culture, OPXV can develop drug-resistant with mutations in F13L or E9L (DNA polymerase), respectively [78,79].

## Targets of neutralizing antibodies

Antibody responses generated against VACV are critical for protection against smallpox [80]. In fact, vaccinia immune globulin (VIG) isolated from plasma of vaccinees, is an effective treatment for smallpox and smallpox vaccine complication [81]. OPXV produces two forms of virions with distinct surface antigens, and antibodies against both forms are required for optimal protection [82,83]. The mature virion (MV) has a single membrane embedded with more than 20 proteins, while the enveloped virion (EV) consists of a MV with a second outer membrane containing eight unique proteins. Seven MV proteins (A13 [84], A17, A27, A28, D8, H3, L1) and two EV-specific proteins (A33 and B5 [84,85]) are known neutralization targets. Anti-B5 antibodies are the dominant EV neutralizing antibodies, while none of the MV neutralizing antibodies are individually required for MV neutralization or dominant in all vaccinated individuals. Instead, the highly redundant neutralizing antibody responses may be a feature of the smallpox vaccine that ensures protection in very different human populations [86]. The antibodies against many of these antigens neutralize in a complement-dependent manner.

Among the MV antigens, H3, A27, and D8 are adhesion molecules that bind glycosaminoglycans (GAGs) on the host cell surface, while L1 and A28 are components of the multi-subunit entry-fusion complex (EFC) that mediates viral fusion with host membranes and virion entry [87]. Human monoclonal antibodies (mAbs) against D8, L1, B5, A33, A27, and H3 were isolated from individuals who had previously been infected with or immunized against OPXV, and these antibodies were able to neutralize multiple OPXV species including MPXV [88]. The mixtures of mAbs targeting both MV and EV had greater neutralizing potential than single mAbs and were more protective than VIG in a mouse model of lethal VACV infection [88].

In recent years, studies of murine or human mAbs elicited with VACV have expanded the knowledge of the protective B cell epitopes [89]. After characterization of five L1 mouse mAbs, three binding sites have been identified with highly variable neutralization capacities [90]. The antibodies with the most potent neutralization activity bind a similar conformational epitope within L1 which mapped to residues 25–34 and 113–131 [90,91]. Four groups of A27 mouse mAbs have been characterized, and the epitope that conferred the greatest protection against disease in mice was pinpointed to a 9-AA linear epitope located in the N-terminus within the heparan binding domain [92,93]. Murine and human antibodies against D8 were found to target a variety of epitopes [94,95]. Chimpanzee-human hybrid and murine antibodies against A33 were found to recognize conformational epitopes [96,97]. Particularly, one murine antibody recognizes the dimer form of A33, allowing it cross-reacts with A33 from other OPXV species [97].

## Conclusion remarks

For a world that is still entangled in the years-long pandemic caused by an exotic animal virus, the mysterious multiple-country outbreak of the rodent-borne MPXV, previously confined to only one area of the world, might be déjà vu of the early days of COVID-19 to some people. Similar questions have arisen: Does the current outbreak reflect a new transmission pattern for MPXV? Has MPXV mutated or have the potential to mutate to be more human transmissible? What can we do to prevent or prepare for a worst-case scenario? We are still too early in the days of the new outbreak to fully answer these questions, but a better appreciation of the MPXV history and biology can provide some clues.

Does the current outbreak reflect a new transmission pattern for MPXV? While the current outbreak might have caught most of the world by surprise, human monkeypox has been re-emerging in Africa for over 20 years. Because previous outbreaks occurred mostly in resource-poor areas, MPXV transmission among the increasingly susceptible and accessible population in Africa has not been well studied, and the disease burden is likely underestimated. What the rest of the world are witnessing today may merely be the same transmission dynamics that has gone on in West Africa for some time but only reaches to a noticeable level when it starts to affect a particularly vulnerable cohort in resource-rich countries. The kind of human or environmental changes that have facilitated the re-emergence of human monkeypox in Nigeria after 39 years of absence might also underlie the current surge of human monkeypox cases in the rest of world. Chief among them is the accumulation of OPXV immune naïve population more than 40 years after the cessation of routine smallpox vaccination. However, the current outbreak does have some unusual features, including the sustained human-to-human transmission among men who have sex with men, that needs to be further studied to understand whether a new transmission pattern has emerged. Regardless, the new reality is that human monkeypox is no longer a rare zoonotic disease and it needs more public health attention.

Has MPXV mutated or have the potential to mutate to be more human transmissible? A common assumption for DNA viruses is that they are genetically stable and have lower evolution potential. While poxviruses do have a slower substitution rate than RNA viruses, they have demonstrated a high recombination potential and can evolve through large structural changes of the genome that result in gene amplification, gene gain, or gene loss. Some of these changes can happen rather quickly in response to selective pressures. While non-synonymous mutations in new viral isolates undoubtedly receive a lot of attention [98], OPXV evolution history informs us that gene loss events in MPXV evolution should also be examined carefully. The MPXV genomes from the current outbreak are still being analyzed. A better understanding of poxvirus evolution mechanisms as well as their gene functions is needed to comprehend the means and consequence of MPXV evolution.

What can we do to prevent or prepare for a worst-case scenario? One lesson COVID-19 has re-taught us is that it is rather unpredictable what virus will emerge to become a significant human pathogen and that it is often too late to develop countermeasures after the fact. It appears prescient today to prepare against some pathogenic viruses even though they currently do not pose a major threat. In this respect, the preparedness for monkeypox is a success story 20 years in the making. Despite the eradication of smallpox in 1970s, the U.S. has prepared for the possibility of smallpox bioterrorism since the 9/11 attack and has taken 20 years to develop and stockpile smallpox vaccines and antivirals, which are also expected to work against monkeypox. Nevertheless, the existing drugs and vaccines still have some drawbacks, including not having been tested in human efficacy trials, some safety concerns, and possible drug resistant mutants. Continued research on poxvirus basic biology is needed for the development of better vaccines and antivirals. For example, studies of VACV neutralizing mechanisms have provided information for the development of nucleic acid-based subunit vaccine for MPXV [99].

In sum, in this post-COVID, more vigilant world, understanding the biology and ecology of the poxvirus family matters ever more.
